# Retrospective Survival Analysis of Cats with Feline Infectious Peritonitis Treated with Polyprenyl Immunostimulant That Survived over 365 Days

**DOI:** 10.3390/pathogens11080881

**Published:** 2022-08-04

**Authors:** Petra Černá, Ashley Ayoob, Caroline Baylor, Erin Champagne, Sandra Hazanow, Robert E. Heidel, Kimberly Wirth, Alfred M. Legendre, Danièlle A. Gunn-Moore

**Affiliations:** 1 Department of Clinical Sciences, Colorado State University, Fort Collins, CO 80523, USA; 2 Small Animal Clinic, The University of Veterinary Sciences Brno, 612 42 Brno, Czech Republic; 3 Critical Care and Internal Medicine Services, Blue Pearl Veterinary Partners, Naples, FL 34112, USA; 4 College of Liberal Arts and Sciences, University of Florida, Gainesville, FL 32601, USA; 5 Independent Researcher, Columbia, MO 65211, USA; 6 Seven Hills Veterinary Hospital, San Francisco, CA 94131, USA; 7 Department of Surgery, University of Tennessee Graduate School of Medicine, Knoxville, TN 37920, USA; 8 Wisconsin Veterinary Referral Center, Waukesha, WI 53188, USA; 9 Small Animal Clinical Sciences, College of Veterinary Medicine, University of Tennessee, Knoxville, TN 37996, USA; 10 The Royal (Dick) School of Veterinary Studies and The Roslin Institute, University of Edinburgh, Easter Bush Campus, Midlothian EH25 9RG, UK

**Keywords:** FCoV, coronavirus, FIP, PI, PPI, innate immune response

## Abstract

Feline infectious peritonitis (FIP) remains a major diagnostic and treatment challenge in feline medicine. An ineffective immune response is an important component of FIP pathophysiology; hence treatment with an immune stimulant such as Polyprenyl Immunostimulant™ (PI), which enhances cell-mediated immunity by upregulating the innate immune response via Toll-like receptors, is a rational approach. Records of cats with FIP treated with PI orally for over 365 days were retrospectively studied. Of these cats (n = 174), records were obtained for n = 103 cats with appropriate clinical signs and clinical pathology. Of these, n = 29 had FIP confirmed by immunohistochemistry (IHC) or reverse transcription polymerase-chain-reaction (RT-PCR). Most of the cats (25/29; 86%) had non-effusive FIP, and only 4/29 cats (14%) had effusive FIP. The mean survival time (MST) was 2927 days (eight years); with 55% of the cats (16/29) still being alive at the time data collection, and 45% (13/29) having died. A persistently low hematocrit plus low albumin:globulin (A:G) ratio, despite treatment, was a negative prognostic indicator. It took a mean of ~182 days and ~375 days, respectively, for anemia and low A:G ratio to resolve in the cats that presented with these laboratory changes. This study shows that PI is beneficial in the treatment of FIP, and more studies are needed to establish the best protocols of use.

## 1. Introduction

Feline infectious peritonitis (FIP) has been a challenge for veterinarians and a devastating disease for cats for over half a century; however, despite marked efforts and many theories, its pathogenesis is still not fully elucidated [1,2]. Clinically, FIP presents in two forms: the effusive (wet) form, which is characterized by fibrinous granulomatous serositis, with protein-rich effusions in body cavities, and the non-effusive (dry) form with phlebitis, serositis and granulomatous inflammatory lesions in several organs (typically liver, kidney, spleen, leptomeninges, and eyes) [3]. The effusive form is more common, typically affecting ~75% of cases, while the non-effusive is seen in ~25% of cases; neurological involvement is typically seen in ~30–40% of cats presenting with non-effusive form [2,4]. The typical clinicopathological changes have been reviewed extensively [5,6]. Of note, 40–90% of cats with FIP present with a low albumin:globulin (A:G) ratio [6,7,8], where hypoalbuminemia is potentially a negative acute phase protein response, and hyperglobulinemia is due to increased immunoglobulins [2,6,7,8,9,10]. Non-regenerative anemia is also common, being attributed to chronic inflammation [6,7,8]. One study reported 67% of cats having mild to moderate anemia at first presentation, with 100% of cats being anemic before death [7].

Feline infectious peritonitis is believed to arise when mutations in a common enterocyte infection (feline enteric coronavirus: FECV) changes its cell tropism, enabling FIP viruses (FIPV) to replicate in monocytes and macrophages [2,11]. However, one study showed that the virulence of different feline coronavirus (FCoV) strains when infecting monocytes did not affect viral replication; rather, the monocytes themselves were responsible for controlling viral replication [12]. Immune responses within individual cats play an important role; effusive FIP results from rapid widespread vasculitis as the cats fail to mount T cell immunity despite a vigorous B cell response [13]. However, cats with non-effusive FIP have a cellular response that is partially effective, managing to contain the FIPV to a relatively small number of macrophages in a few focal sites within specific target organs [13]. Cats that resist FIPV are believed to mount a vigorous cell-mediated immune response and overcome the negative effects of antibodies to these FCoV (including FECV and FIPV) [13]. Cell-mediated immunity therefore plays an important role in controlling or eliminating the mutated virus. Cats in the terminal stages of FIP have severe depletion of the CD4^+^ and CD8^+^ T-lymphocytes necessary for mounting cell-mediated immunity (CMI) [14,15]. Several authors suggested that pathology may be associated with the onset of something similar to a cytokine storm, coupled with Th-1 pathways dysregulation [15,16,17], such that proinflammatory responses lead to systemic inflammation and clinical FIP in cats [13,16].

With immune dysregulation being a major component of the pathophysiology of FIP, it is rational to try and treat this disease with an innate immunity stimulant. Polyprenyl Immunostimulant™ (PI) is believed to correct aberrant immune response via integrins (patent claim pending) and maintain CMI by upregulating innate immunity via Toll-like receptors [18], and thus may be of benefit in diseases involving suppression of CMI. PI is regulated and licensed by the U.S. Department of Agriculture [18] to treat disease caused by feline herpesvirus (FHV-1, also known as feline rhinotracheitis virus) in cats over eight weeks of age and is safe to give to kittens from two days of age [19]. In a pilot study and a larger field study PI has also shown promise as a treatment for cats with FIP [20,21].

The current study is the first to look at survival analysis of cats with FIP that have been treated with PI and survived over 365 days. It also proposes blood parameters that may be useful for monitoring the response to treatment.

## 2. Results

A total of n = 174 cats were identified for whom PI had been continuously purchased for over 365 days for the treatment of FIP according to the sales records; patient records were available for n = 103 of them. A diagnosis of FIP was confirmed by routine commercial RT-PCR or IHC in n = 29 cats; the data from these cats were used in this paper.

All of the 29 cats included in this study were diagnosed and treated by their own veterinarians. No side effects from PI treatment were noted by the owners or veterinarians.

### 2.1. Signalment and Clinical Signs

All 29 cats had clinical signs consistent with FIP at the time of the initial veterinary visit (Table 1); n = 19 were male and n = 10 female, n = 13 were Domestic Shorthair, n = 4 Persian, n = 3 Domestic longhair, n = 3 Sacred Birman, n = 2 Siamese, n = 1 Somali, n = 1 Abyssinian, n = 1 Maine Coon, and n = 1 Norwegian Forest Cat. The median age at diagnosis was 39 months (range 3–141 months). The main presenting signs were weight loss (n = 16 cats), inappetence (n = 12), pyrexia (n = 6), vomiting (n = 6), lethargy (n = 5), diarrhea (n = 5), uveitis (n = 2), dyspnea (n = 2), cough (n = 1), ataxia (n = 1), lameness (n = 1), straining to urinate (n = 1), respiratory signs (n = 1), and stunted growth (n = 1). Non-effusive FIP was diagnosed in 25/29 cats (86%) and effusive FIP was found in 4/29 cats (14%). Possible causes of the high prevalence of cats with non-effusive FIP have been discussed in the discussion.

### 2.2. Diagnosis

The diagnosis of FIP was confirmed in n = 22 cats by positive IHC (lymph nodes, abdominal masses, intestines, mediastinal mass, or eye) or by positive RT-PCR (pleural effusion, lymph node aspirate or bronchoalveolar lavage fluid) for FCoV in n = 7 cats (Table 1). Serum protein electrophoresis was performed in 7/29 cats and showed polyclonal gammopathy in all cases. Serum alpha-1-acid glycoprotein was only measured in 2/29 cats and was high in both cases. Cytology results were available in 11/25 cats; in most cases showing pyogranulomatous 4/12 or mixed 3/12 inflammation, reactive lymphoid hyperplasia in 2/12, lymphocytic inflammation in 1/12 and in two cases were non-diagnostic. Neutrophil counts were available for 22/29 cats; 59% of these cats presented with neutrophilia, and mean neutrophil count was 15.6 × 10^3^/µL (±9.1).

### 2.3. Treatment

Data for the dose of PI were available for 25/29 cats (Table 2). A total of 8/25 cats stayed on the full dose from the start of treatment (3 mg/kg three times a week or 3 mg/kg every other day administered orally), while 11/25 were weaned to a maintenance dose (ranging from 3 mg/kg once per week to 3 mg/kg twice a week) starting at around a year after diagnosis; however, one cat relapsed when the PI dose was decreased, so it was re-started on a higher dose of PI. In the other seven cats (28%), the PI was discontinued after 10 to 36 months of treatment (when the A:G ratio and HCT values normalized); four of these seven cats are currently alive, although one of them relapsed and was treated again with the full dose and stabilized. In 6/25 cats, the PI was tapered down to a maintenance dose and eventually stopped; all of the six cats in which PI was stopped were alive without PI for at least 1 year (Table 2). Prednisolone was concurrently administered in 2/25 cats; no other medications were administered long-term.

### 2.4. Clinicopathological Findings

**Figure 1:**
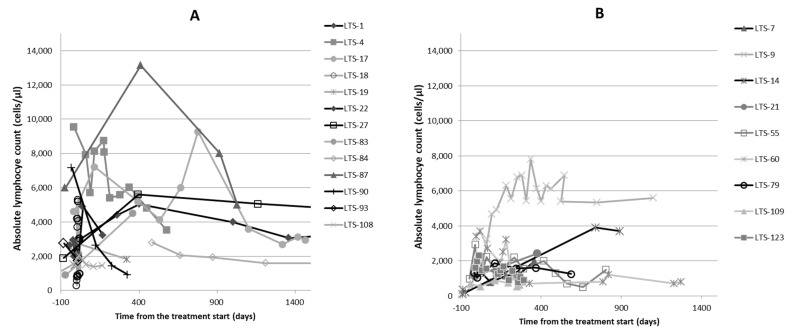
Changes in the lymphocyte counts over time in the 21 cats whose follow-up blood work was available. (**A**) shows changes in the lymphocyte counts for Alive cats and (**B**) shows changes in the lymphocyte counts for Dead cats over time (note that the last measurement is not at death). LTC 1 = cat number 1, etc.

**Figure 2:**
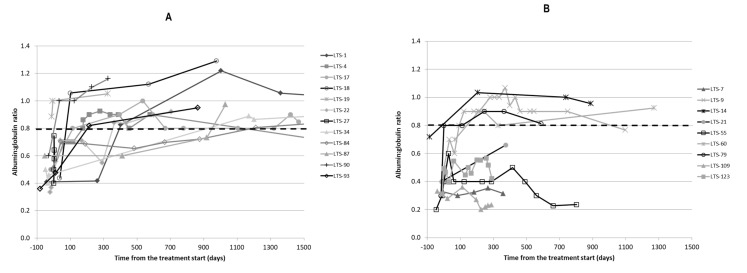
Changes in A:G ratio over time in the 21 cats whose follow-up blood work was available. (**A**) shows changes in albumin:globulin ratio for Alive cats and (**B**) shows changes in albumin:globulin ratio for Dead cats over time (note that the last measurement is not at death). Dotted line shows albumin:globulin ratio of 0.8 (normal). LTC 1 = cat number 1, etc.

Initial and follow-up bloodwork was available for 21/29 cats. A total of 7/21 cats (33%) presented with lymphopenia and in 6/7 cats (85%) the lymphopenia resolved on PI treatment; however, in 3/6 of these cats, the lymphopenia returned before death. The lymphocyte counts for both the Alive and Dead groups are shown in Figure 1. A total of 18/21 cats (86%) had low A:G (≤0.8) at the time of diagnosis; their mean A:G was 0.49 (±0.11) at the time of diagnosis. Figure 2 shows the changes in A:G over time. In 12 cats (67%), the A:G improved to ≥0.8 on treatment with PI. The ratio increased to a mean of 0.88 (±0.11, range 0.8–1.1) in a mean of 375 (±481) days. The mean baseline A:G for the Alive and Dead groups was 0.58 (±0.25) and 0.48 (±0.26), respectively. The mean last available A:G for the Alive and Dead groups was 0.90 (±0.16) and 0.56 (±0.32), respectively. There was a significant difference between the Alive and Dead groups for the last available A:G (*p* = 0.024), while there was no significant difference between the groups at baseline A:G (*p* = 0.37) prior to starting the PI treatment. There was also a significant difference between the Alive and Dead groups for the improvement in A:G over time (*p* = 0.001).

**Figure 3:**
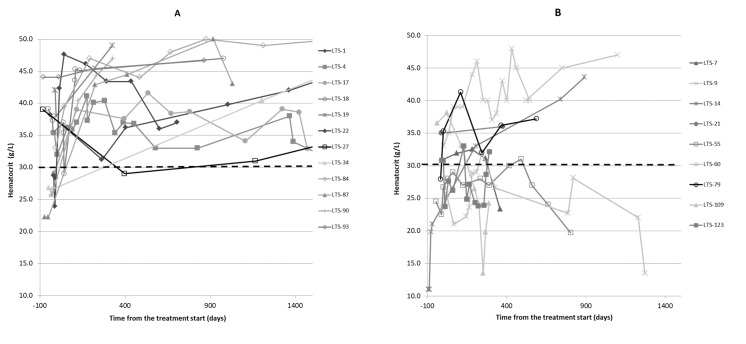
Changes in hematocrit over time in the 21 cats whose follow-up blood work was available. (**A**) shows changes in hematocrit (HTC) for Alive cats and (**B**) shows changes in HTC for Dead cats over time (note that the last measurement is not at death). Dotted line shows HTC of 30% (normal). LTC 1 = cat number 1, etc.

Anemia (HCT ≤ 30%) at the time of diagnosis was reported in 8/21 cats (38%), with one cat requiring a blood transfusion (LTS-14). It took a mean of 181.5 (±120.7) days for the hematocrit value to normalize. Figure 3 shows the change in HCT with time. The mean baseline HCT for the Alive and Dead groups was 32.8 (±6.3) and 28.4 (±8.6), respectively. The mean most recent HCT for the Alive and Dead groups was 42.1 (±5.4) and 29.9 (±11.8), respectively. There was no difference between the Alive and Dead groups for the change in HCT over time (*p* = 0.14), but there was a significant difference between the Alive and Dead groups for most recent HCT (*p* = 0.023). There was no significant difference between the groups in baseline HCT (*p* = 0.18) prior to starting PI.

### 2.5. Survival Time

**Figure 4:**
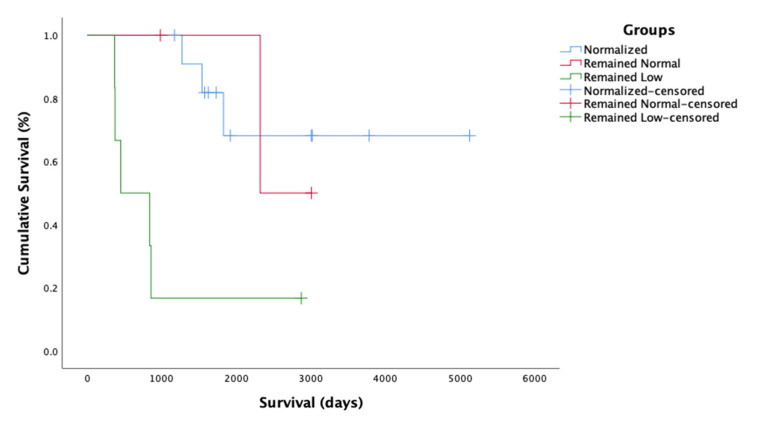
Survival curve for albumin: globulin ratio groups. Censored = alive on 19 June 2020.

The MST was 2927 days (eight years), ranging from 365 to 5126 days (1–14 years), with the longest surviving cat still alive and being treated with PI at the time of publication (5126 days). A total of 55% (16/29) cats were still alive at the time of data collection, and 45% (13/29) cats died. Further analysis was performed on 21/29 of the cases with sufficient follow-up data related to A:G while on the PI treatment. The cats were subdivided into groups based on changes in their A:G while being treated with PI: (1) Normalized, i.e., A:G increased from low (<0.8) to normal (≥0.8) (n = 12); (2) Remained normal, i.e., the A:G was normal at the beginning of PI treatment and stayed that way (n = 3); and (3) Remained low, i.e., the A:G remained low (<0.8) (n = 6). The following MSTs with 95% CIs for the groups were: Normalized (*MST* = 4000, 95% CI 2918–5082), Remained normal (*MST* = 2664, 95% CI 2187–3140), and Remained low (*MST* = 960, 95% CI 257–1662). There was a statistically significant difference in survival times among the three groups, χ^2^(2) = 11.0, *p* = 0.004. The survival curve for the A:G analysis is presented in Figure 4.

**Figure 5:**
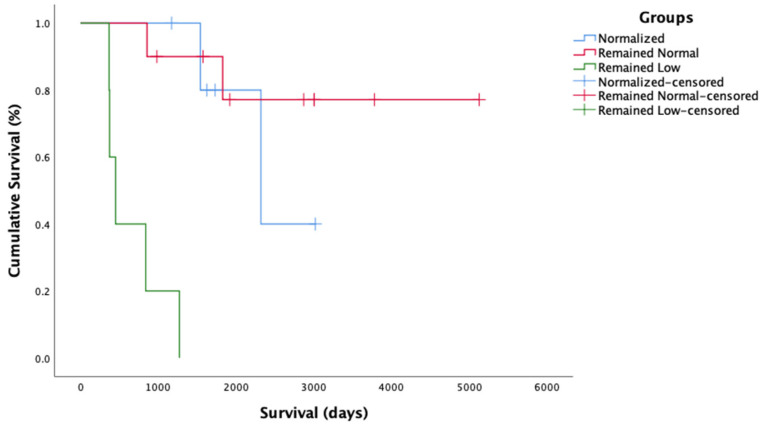
Survival curve for hematocrit groups. Censored = alive on 19 June 2020.

The cats with available data were also subdivided into groups based on changes in their HCT while on the PI treatment: (1) Normalized, i.e., HCT increased from low (<30%) to normal (≥30%) (n = 6); (2) Remained normal, i.e., HCT remained within normal limits from the beginning of the treatment (n = 10); and (3) Remained low, i.e., HCT remained low or decreased (<30%) (n = 5). The MSTs with 95% CIs for each group were as follows: Normalized (*MST* = 2444, 95% CI 1890–2999), Remained normal (*MST* = 4275, 95% CI 3221–5320), and Remained low (*MST* = 661, 95% CI 317–1005). There was a statistically significant difference (*χ*^2^(2) = 25.2, *p* < 0.001) in survival time among the groups depending on the resolution or persistence of anemia. Cats whose anemia did not resolve or who became anemic during the treatment had the shortest survival time. The survival curve for the HCT analysis is presented in Figure 5. Cats with unresolved anemia and low A:G (n = 3) had the shortest survival times and lived 553 (±251) days on the treatment.

**Figure 6:**
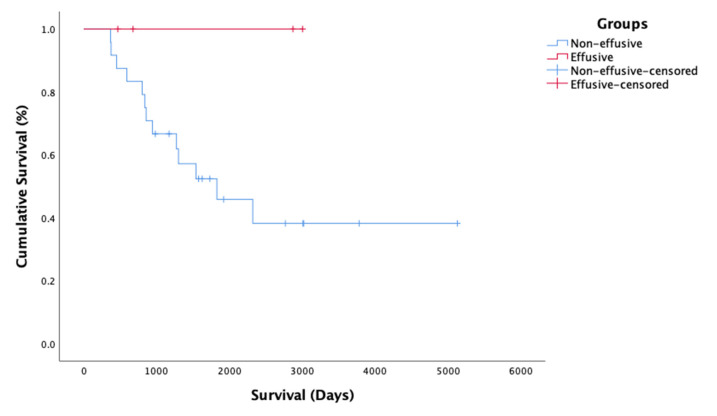
Survival times for the cats with Non-effusive (blue, n = 25) and Effusive (red, n = 4) FIP. The results of the Kaplan–Meier analysis show no statistically significant differences in survival between the groups (*p* = 0.14); however, the power of the effusive group is very low due to low number of patients. Censored = alive on 19 June 2020.

The mean survival time of all n = 29 cats was 2927 (95% CI 2091–3763). When analyzing the survival of cats with Effusive (n = 4) and Non-effusive (n = 15) FIP, there was no significant difference between the groups (*p* = 0.14). The survival curve is presented in Figure 6. MST could not be calculated for the Effusive group, as all four cats in this group were still alive.

## 3. Discussion

This study adds important information about the successful treatment of FIP with PI; it shows that PI can be useful in the management of this disease. All of the cats had clinical signs consistent with FIP when they started PI treatment, and all had been treated with PI continuously for at least 365 days. The MST was 2927 days, with the longest surviving cat still alive and being treated with PI at 5126 days. Interestingly, the median age at FIP diagnosis was 39 months (ranging from 3 to 141 months). This is older than usually expected, as FIP typically affects young cats (<2 years of age) (18). However, 6% of non-effusive forms of FIP (e.g., neurological) are seen in middle-aged cats of 2–8 years of age, and can also occur in 2% of older cats (>8 years) [22]. The older age of the cats in the current study reflects that over 80% of the cats had non-effusive FIP.

The cats that were most likely to respond positively to PI treatment were those in which the A:G ratio and/or HCT remained normal or returned to normal with treatment. Cats with unresolved anemia and low A:G had the shortest survival time on PI treatment. There were no adverse effects of PI treatment noted by the owners or the veterinarians.

Since PI is an immunomodulatory drug that enhances CMI by upregulating innate immunity via Toll-like receptors two and four pathways [18], it may be of benefit in diseases like FIP that cause dysregulation in cellular immunity [14,15]. PI is likely more effective in cats with non-effusive FIP, as these cats are believed to have a partially effective CMI, while cats with the effusive form fail to mount T cell immunity [13]. Further studies are needed to fully assess the benefits of PI in the effusive and non-effusive forms of FIP. Effusive FIP is typically more common than non-effusive FIP at diagnosis (~75% verses ~25%) [2,4,6]. The reason for the low number of effusive cases in the current study is probably because PI was only recommended for non-effusive cases [19]; however, other factors may also have been involved, such as effusive cases being more acute and severe, so owners elected for euthanasia instead.

Changes in serum chemistry and hematology are common in cats with FIP. A low A:G ratio (<0.8) is thought to mainly result from hyperglobulinemia due to increased antibody globulins, acute phase proteins and complement factors [2,9,10,23]; it is typically seen in about 60% of cases [6,7,8], and was present in 86% (18/21) of cases in the current study. Non-regenerative anemia (HTC < 30%) is usually attributed to inflammation and is typically seen in 66–100% of FIP cases [6,7,8]; it was seen in 38% (8/21) cats in this study. One third of the cats presented with lymphopenia and in 85% of these cats, the lymphopenia resolved on PI treatment. The current study showed that normalization of these abnormalities was prognostically important. A combination of A:G ratio and HTC was reported as a good indicator of treatment progress in another study, where cats with FIP were treated with the antiviral nucleoside analog GS-441524 [24]. In the current study, cats with a normal A:G at the time of starting PI treatment had the best prognosis, followed by those whose A:G ratio normalized on treatment, while those whose A:G started low and remained low had the shortest survival times. Likewise, cats with a normal HCT at the time of starting treatment had the best prognosis, followed by those that normalized on treatment, while those whose HCT remained low had the shortest duration of survival. This is consistent with a previous study that reported 100% of cats with FIP becoming anemic before death [7]. Data on hematological abnormalities for cats that did not survive to 365 days is not available, and it is therefore difficult to understand if non-anemic cats respond better to PI treatment, but persistent anemia in cats was suggestive of poorer outcome.

The PI appeared to take time to work. Cats that started with anemia took a mean of ~182 days to normalize, whereas a low A:G ratio took a mean of ~375 days for this to resolve. However, the retrospective nature of this study means there was significant variation between the timing of repeat blood examinations, with some cats not being re-examined for many months. The cats with unresolved low A:G and anemia (n = 3) had the shortest survival, living a mean of 553 days on the PI treatment. Unfortunately, our study population was not large enough to analyze HCT and A:G values co-dependently. Nor are we able to comment on the effect of low A:G ratios or anemia in cats treated with PI that did not live to a year on treatment. Another possible factor that may influence the response to treatment is the time elapsed from infection to the beginning of clinical signs and the start of treatment. In a field study (i.e., a non-experimental study), this time cannot be standardized and is usually unknown, but it may influence the response (and the time of the response) to treatment.

Many of the cats (18/25; 72%) in the current study stayed on PI from diagnosis to death or were still alive at the end of the study (Table 2). In the other seven cats (28%), the treatment was discontinued after 10 to 36 months of treatment (when the A:G ratio and HCT values normalized); four of these seven cats are currently alive, although one of the seven relapsed and was treated again with the full dose and stabilized. One of these cats survived over 365 days despite the treatment being discontinued after 10 months. Of note, in 4/8 cats on the full dose throughout treatment, the HCT and A:G never normalized. It is likely that the full dose was maintained in these cats as they were particularly ill, while it was reduced in the cats that improved. Further studies are needed to evaluate the implications of decreasing or even discontinuing PI in cats with FIP. It has been previously reported that concurrent administration of corticosteroids significantly shortens the MST of cats with FIP when treated with PI [21]. While only 2/25 cats in the current study were treated with PI plus prednisolone, which was too few to allow statistical survival analysis, both lived for less than the MST.

It has been suggested that a lack of CMI to eliminate cells infected with FCoV (FIPV) allows for further virus production and permits the virus to persist in tissue macrophages [25,26]. This was also supported by a study where experimental infection with FIPV resulted in only temporary clinical disease in a number of cats; these cats were found to be free of lesions typical of FIP on post-mortem examination and it was suggested that in these cats, the CMI was able to overcome the infection [15]. While the method by which PI induces immune-modulation still has yet to be completely elucidated, it was recently shown that PI upregulated the expression of CD11b on monocytes in cats that were treated with 0.5 mg/mL PI twice daily for 14 days (patent claim pending).

Successful treatment of FIP with new antiviral compounds, e.g., nucleoside analog GS-441524 and 3C-like protease inhibitor, is looking very promising [24,27]; however, they are not universally effective. It has been suggested that antiviral drugs can act synergistically with immunomodulatory treatments to improve patient survival in a number of viral diseases, such as human influenza virus, hepatitis C virus and human immunodeficiency virus (HIV) [28,29,30] and combinations of these antiviral drugs with immune-modulators, such as PI, may potentially improve the long-term treatment outcomes for cats with FIP [31].

There are several limitations of this study. First, this is a retrospective study, with all of the potential biases that confers. Another limitation is that although all of the cases had a positive IHC or RT-PCR for diagnosis of FIP, additional diagnostics (e.g., serum protein electrophoresis, serum or fluid alpha-1-acid glycoprotein, and cytology) were not performed in all of the cases and the incomplete clinicopathological profile of these cats makes it difficult to evaluate the inflammatory and immune responses of these cats. Follow up of these tests, such as resolution of polyclonal gammopathy or increased levels of acute phase proteins during treatment with PI, could not be evaluated.

Importantly, we were unable to gain case details for all of the cats that were given PI to treat FIP, and so could not determine what percentage of all cats that were treated with PI for FIP survived. This was because the company that makes PI lacks case information on many cases; veterinarians rarely name the patient the PI is for, or even whether it is to treat FHV-1 (for which it is licensed) or FIP. It was not possible to follow up all of the PI orders, which would be needed to determine this, due to incomplete records, incorrect diagnosis, veterinarians moving on, etc. Because of this, we elected to assess the cats where it was clear that a named veterinarian was treating a specific patient for FIP, and had been doing so for a reasonable period of time, which was set at over 365 days. Another limitation is a lack of comparator group (e.g., patients with corticosteroids only or antiviral drugs)—in the future, a prospective controlled study comparing PI, PI plus remdesivir or GS-441524, and remdesivir or GS-441524 alone would be ideal. Other limitations of this retrospective study were the lack of a standardized dose and a standardized monitoring protocol, with each patient’s care being determined by the veterinarian in charge of that case.

## 4. Materials and Methods

VetImmune^®^ (Sass & Sass Inc, Oak Ridge, TN, USA), the distributor of PI, searched their sales records from 2012–2019 for cats treated with PI continuously for at least 365 days where their veterinarians listed FIP as the indication for the order. Additional cases came from prior to pre-license approval (2006–2011). The cats’ medical records were obtained from the veterinarians to assess the data under conditions of privacy where no personal information was disclosed. All records were obtained with the consent of the cats’ owners.

A full case record with details of the entire diagnostic workup was mandatory for each case inclusion. The records contained copies of physical examinations, test results and follow-up veterinary visits during the PI treatment. The records had to include the date of the start of the treatment, follow-up and death of the cat where that occurred. Where follow-up data were missing, these were obtained by correspondence with the patient´s veterinarian or the owner.

All cases were reviewed by a feline medicine specialist (DGM) to confirm the standard of diagnostics. The records were analyzed by DGM and PC. All cats had clinical signs consistent with FIP and a thorough diagnostic work-up that excluded other disease (e.g., FIV, FeLV and toxoplasmosis). The FIP diagnosis was confirmed by routine commercial confirmatory assays, i.e., histological FCoV immunostaining (i.e., immunohistochemistry [IHC]) or by FCoV reverse transcription polymerase-chain reaction (RT-PCR) (Table 1). The cats were then divided into Alive at the time of data collection or Dead.

The recommended starting dose of PI was 3 mg/kg orally three times a week up to 2019, after which it changed to 3 mg/kg orally once a day for the first two weeks and then 3 mg/kg orally every other day. Cats above 5 kg were to receive a total dose of 15 mg; however, dose changes in individual cats in the study were at the discretion of the veterinarians.

Independent samples t-tests were used to compare the changes in albumin to globulin ratio (A:G) and hematocrit (HCT) between the Dead and Alive cats. Means and standard deviations were reported and interpreted for the t-test analyses. Cats were divided into subgroups according to the change in their A:G and HCT values while on PI treatment for further statistical analysis (i.e., normalized, remained normal, and remained low). The subgroups were compared to one another. Kaplan–Meier (KM) analysis was used to compare the A:G subgroups (normalized, remained normal, and remained low) on the “time-to-event” associated with mortality (death). A separate K-M analysis was performed to compare the HCT subgroups (normalized, remained normal, and remained low). The time signature for the K-M analyses was in days from the start of PI treatment to death, or through to 19 June 2020 for the cats that were still alive. The MST (measured in days from the start of the PI treatment) with a 95% confidence interval (95% CI) was reported and interpreted for each group. The log-rank test was used to compare the respective groups (on the time to death). The mean (M) and 95% CI associated with time to death was reported and interpreted for each group. The data for KM analysis were censored, and the date of June 19, 2020 was used for the cats still alive. Statistical significance was assumed at an alpha value of 0.05, and all analyses were performed using SPSS Version 26 (Armonk, NY, USA: IBM Corp.).

## 5. Conclusions

This study adds important data about the successful treatment of FIP with PI; it shows that PI is beneficial in the management of this disease, and reports survival analysis of these cats. The MST was 2917 days (eight years) for those cats that survived to at least 365 days. A combination of resolving anemia and normalization of A:G ratios during the PI treatment appears to be a positive prognostic indicator for survival. This study demonstrates that PI is useful in the treatment of FIP, and a prospective study is needed to investigate the efficacy of decreasing the dose and to see if complete discontinuation of PI could be considered.

## 6. Patents

Patent Application No. 17/343,047 Unpublished (filing date 9 June 2021) (T. Kuritz and J.P. Biggerstaff, applicants).

## Figures and Tables

**Table 1:** Signalment, survival times, and clinical signs of the 29 cats with FIP.

**#**	Breed	Sex	Age at Diagnosis (Years)	Survival Days *	Clinical Signs at Presentation	Diagnosis Confirmed by	Cause of Death (Where Known)
LTS-1	Domestic Shorthair	MC	3.8	Alive 5126	Weight loss	IHC (mesenteric LN)	
LTS-4	Domestic Shorthair	MC	0.4	Alive 3780	Diarrhea	IHC (intestines)	
LTS-6	Domestic Shorthair	FS	3.8	Dead 802	Weight loss, inappetence	IHC (abdominal mass)	Lymphoma
LTS-7	Domestic Longhair	FS	11.8	Dead 374	Weight loss, vomiting, diarrhea	IHC (intestinal mass)	Anemia
LTS-9	Domestic Longhair	MC	3.3	Dead 2035	Weight loss	IHC (abdominal mass	Renal failure
LTS-14	Maine Coon	MC	4.3	Dead 2320	Weight loss	IHC (mediastinal mass)	HCM
LTS-15	Domestic Shorthair	MC	3.1	Dead 943	Weight loss, lethargy	IHC (mesenteric LN)	Unknown
LTS-17	Sacred Birman	FS	0.5	Alive 3020	Straining to urinate, stunted growth	IHC (abdominal mass)	
LTS-18	Domestic Shorthair	MC	3.3	Alive 3002	Inappetence, lethargy, pyrexia, cough	IHC (LN)	
LTS-19	Domestic Longhair	FS	12.8	Alive 3007	Inappetence, vomiting	IHC (abdominal mass)	
LTS-21	Domestic Shorthair	MC	1.8	Dead 856	Inappetence, weight loss, pyrexia, diarrhea	IHC (mesenteric LN)	FIP
LTS-22	Domestic Shorthair	MC	2.2	Alive 1171	Inappetence, weight loss	IHC (LN)	
LTS-27	Persian	FS	4.4	Alive 2870	Vomiting	IHC (liver and LN)	
LTS-29	Siamese	MC	11.3	Dead 1301	Inappetence, weight loss, uveitis, diarrhea, pyrexia	IHC (colon)	FIP
LTS-34	Sacred Birman	MC	0.5	Alive 2767	Pyrexia	IHC (mesenteric LN)	
LTS-55	Domestic Shorthair	MC	6.2	Dead 839	Weight loss	IHC (abdominal LN)	Anemia
LTS-60	Persian	FS	6.9	Dead 1272	Inappetence, weight loss, lethargy, lameness, pyrexia	IHC (popliteal LN)	Renal failure
LTS-79	Siamese	MC	11.2	Dead 1541	Inappetence, weight loss, diarrhea, vomiting	IHC (Ileocolic mass)	Unknown
LTS-83	Sacred Birman	MC	5.8	Alive 2023	Weight loss	IHC (LN)	
LTS-84	Abyssinian	FS	6.7	Alive 1920	Respiratory signs, lethargy	RT-PCR (BAL fluid)	
LTS-87	Domestic Shorthair	MC	0.3	Alive 1731	Ataxia	RT-PCR (LN)	
LTS-90	Domestic Shorthair	MC	0.5	Alive 1625	Pyrexia	RT-PCR (LN)	
LTS-93	Norwegian Forrest Cat	MC	1.4	Alive 1575	Inappetence	RT-PCR (LN)	
LTS-98	Domestic Shorthair	FS	4.3	Dead 591	Inappetence, weight loss, uveitis	IHC (eye globe)	Unknown
LTS-108	Persian	FS	0.6	Alive 982	Vomiting	IHC (mesenteric LN)	
LTS-109	Somali	FS	9.7	Dead 368	Inappetence, vomiting	RT-PCR (LN)	Anemia
LTS-123	Domestic Shorthair	MC	0.6	Dead 451	Inappetence, weight loss	IHC (abdominal mass)	Anemia
LTS-146	Persian	MC	3.0	Alive 677	Weight loss, dyspnea	RT-PCR (pleural effusion)	
LTS-149	Domestic Shorthair	MC	0.3	Alive 469	Lethargy, dyspnea	RT-PCR (pleural effusion)	

* ALIVE means the cat was alive on 19 June 2020 (* number of days since PI started); Y—years; M—months; MC—male castrated; FS—female spayed; RT-PCR—reverse transcription polymerase chain reaction; IHC—immunohistochemistry; BAL—bronchoalveolar lavage; LN—lymph node.

**Table 2:** PI dosing for 25 of the 29 cats with FIP.

#	PI Dose	Note
LTS-1	Decreased to maintenance dose *	
LTS-4	Decreased to maintenance dose *	
LTS-7	Remained on full dose	
LTS-9	Decreased to maintenance dose *	
LTS-14	Discontinued	Alive off PI for over 1 year
LTS-15	Discontinued	Alive off PI for over 1 year
LTS-17	Decreased to maintenance dose *	
LTS-18	Full dose again after relapse	Relapsed when PI stopped or decreased
LTS-19	Decreased to maintenance dose *	
LTS-21	Remained on full dose	Concurrent treatment with prednisolone (from 5 mg every 12 h to 2.5 mg every over day)
LTS-27	Discontinued	Alive off PI for over 1 year
LTS-34	Discontinued	Alive off PI for over 1 year
LTS-55	Remained on full dose	
LTS-60	Remained on full dose	Concurrent treatment with prednisolone (1.25 mg every 24 h)
LTS-83	Remained on full dose	
LTS-84	Decreased to maintenance dose *	
LTS-87	Discontinued	Alive off PI for over 1 year
LTS-90	Discontinued	Alive off PI for over 1 year
LTS-93	Decreased to maintenance dose *	
LTS-98	Remained on full dose	
LTS-108	Decreased to maintenance dose *	
LTS-109	Remained on full dose	
LTS-123	Remained on full dose	
LTS-146	Decreased to maintenance dose *	
LTS-149	Decreased to maintenance dose *	

* When the PI was decreased, reduction to maintenance dose was done at around a year after diagnosis if the cat was clinically stable.

## Data Availability

Not applicable.
